# Comparison of the Efficacy of Different Exercise Modes on MCI Adults: A Network Meta‐Analysis

**DOI:** 10.1002/brb3.70734

**Published:** 2025-08-21

**Authors:** Changjiang Qiu, Jiaxin Han, Yao Xie, Lu Liu, Yufeng Zeng, Liangyi Xiao, Shanshan Zeng, Le Xie, Dahua Wu

**Affiliations:** ^1^ Hunan University of Chinese Medicine Changsha China; ^2^ Hunan Provincial Hospital of Integrated Traditional Chinese and Western Medicine (The Affiliated Hospital of Hunan Academy of Traditional Chinese Medicine) Changsha China; ^3^ Department of Neurology Hunan Provincial Hospital of Integrated Traditional Chinese and Western Medicine (The Affiliated Hospital of Hunan Academy of Traditional Chinese Medicine) Changsha China; ^4^ Department of Acupuncture and Moxibustion Rehabilitation Changsha Hospital of Traditional Chinese Medicine (Changsha No. 8 Hospital) Changsha City China

**Keywords:** cognition, MCI, multicomponent exercise mode, network meta‐analysis

## Abstract

**Background:**

Mild cognitive impairment (MCI) is considered a preclinical or prodromal stage of Alzheimer's disease (AD). MCI patients have an annual probability of progression to dementia of 10%–15%, far higher than the normal elderly (∼1%–2%). We conducted a network meta‐analysis (NMA) on the efficacy of various exercise modes on MCI adults.

**Methods:**

A computer search was conducted in Embase, PubMed, the Cochrane Library, and Web of Science for RCTs on the effects of exercise training on MCI patients from their inception to February 22, 2025. Two reviewers were independently responsible for study screening, data extraction, and quality assessment. RevMan 5.4, Stata15, and R4.4.1 were utilized for data analysis.

**Results:**

Thirty‐five studies with 2717 patients were included. The NMA results revealed that combination training (CT) performed best in increasing the MoCA and MMSE scores of MCI patients, with SUCRA of 99.43% and 87.04%, respectively. Resistance training and sensorimotor training were the second‐best interventions for increasing MMSE and MoCA scores, respectively.

**Conclusion:**

CT is the best intervention to improve MOCA and MMSE scores in patients with MCI, which can effectively alleviate the disease progression of MCI. However, a variety of double‐blind, large‐sample clinical trials are still needed to further verify this conclusion.

## Graphical abstract

Combination training (CT) significantly improves cognitive function in individuals with mild cognitive impairment (MCI), outperforming other exercise modalities such as aerobic or resistance training. This network meta‐analysis identifies CT as the most effective intervention for enhancing MoCA and MMSE scores, offering a promising non‐pharmacological approach for MCI management.

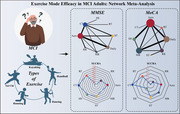

## 1 Introduction

Mild cognitive impairment (MCI) is considered a preclinical or prodromal stage between brain aging and early dementia (Christa Maree Stephan et al. 2013), and this neurodegenerative disease (Petersen et al. 2014) presents a mild decline in cognitive function (e.g., deficits of memory, calculation, or verbal expression) (Pei et al. 2020) but roughly normal activity of daily living (ADL). Due to persistent factors for nerve injury, however, MCI can progress to irreversible CI (Lissek and Suchan 2021). MCI patients over 65 years old have a high annual probability of progression to dementia, and about 10%–15% of them will develop Alzheimer's disease (AD) each year (Misra et al. 2009), far higher than the normal elderly (∼1%–2%) (Bischkopf et al. 2002). To sum up, MCI not only seriously affects the quality of life (QoL) of the elderly but also imposes a great economic burden on the healthcare system (Patterson 2018; J. Duan et al. 2020).

Medications for MCI have not made important breakthroughs and may even fail, leading to adverse effects or drug resistance (Chan et al. 2013). Cognitive interventions, or cognitive training have been widely explored in the treatment of MCI (Fitzpatrick‐Lewis et al. 2015). However, the great heterogeneity in the clinical manifestations of MCI of diverse cognitive domains has yielded complex interventions, including single‐domain (such as memory enhancement) and multi‐domain cognitive training, and integrated intervention combining exercise or diet regulation. In general, single‐domain cognitive training is weak in the intervention effect (Ströhle et al. 2015). Multi‐domain cognitive training and integrated intervention are more effective and can better generate transfer effects of interventions (Hill et al. 2017), but their implementation is more difficult due to diverse and complex intervention protocols. As shown by epidemiologic data, regular physical activity is linked to a lower incidence of dementia (Blüchel et al. 2013). Exercise has grown in significance as a promising non‐medication means (Huang et al. 2020), which can prevent cognitive decline in CI individuals. Meanwhile, several RCTs have documented the benefits of exercise for cognitive function in CI patients (Mollinedo Cardalda et al. 2019; Langoni et al. 2019a). Previously, it was found that different types of exercise may work through different molecular mechanisms, producing different treatment effects (Landrigan et al. 2020; L. Zhang et al. 2020; Shu et al. 2020). Therefore, the type of exercise should be taken into account when exercise is adopted by clinical professionals to prevent or delay cognitive decline. However, the most effective exercise in preventing and delaying cognitive decline or ameliorating CI remains unclear.

A meta‐analysis (Gates et al. 2013) revealed that exercise can ameliorate cognitive function in patients with MCI; specifically, aerobic training (Aero) significantly improves cognitive function, but the reliability of the conclusions remains to be demonstrated due to great heterogeneity, whereas combination training (CT) displays no significant effect (Gates et al. 2013). Dan Song argued that Aero is more effective than other exercises in improving the overall condition of patients with MCI (Song et al. 2018), and Han et al. demonstrated that two or more combinations of exercise interventions can improve memory in older populations (Reina‐Gutiérrez et al. 2023). Multicomponent exercise mode also performs better than Aero in improving cognitive performance in patients with MCI (Bisbe et al. 2020). Besides, a previous network meta‐analysis (NMA) explored the efficacy of different types of exercise on overall cognition in adults with MCI, but it included studies published until 2018 and did not take into account multicomponent exercise modes (S. Wang et al. 2019). Therefore, we conducted an NMA to compare the relative efficacy of different types of multicomponent exercises and to determine the optimal exercise intervention for protecting cognitive function in patients with MCI.

## 2 Methods

This NMA was carried out following the Preferred Reporting Items for Systematic Reviews and Meta‐Analyses (PRISMA)‐NMA and registered with PROSPERO (CRD570034).

### 2.1 Search Strategy

We searched Embase, PubMed, Web of Science, and the Cochrane Library from their inception to February 22, 2025 (Appendix). To ensure full coverage of eligible studies, we scrutinized the references of systematic reviews published in the last 3 years (Duan et al. 2018; de Souto Barreto et al. 2018; Liang et al. 2018; Bruderer‐Hofstetter et al. 2018) and manually searched for additional relevant studies. We used medical subject headings plus free words including “cognition,” “cognitive decline,” “exercise,” “Tai Chi,” and “yoga.” Experts of CI and sports health reviewed the search strategy for comprehensiveness and accuracy.

### 2.2 Study Screening

After duplicate publications were removed using EndNote 21, potentially eligible studies were identified by reading the title and abstract by two reviewers (QC and HJ). Finally, eligible studies were independently assessed and included by the two reviewers. Discrepancies were resolved through discussion or consultation with a third reviewer (XY) if necessary.

### 2.3 Eligibility Criteria

Inclusion criteria: (1) Population: adult patients diagnosed with MCI (Petersen et al. 2014, US 2013). (2) Indicators: overall cognition and any cognitive domains (verbal fluency, cognitive speed, delayed recall, immediate recall, executive function, working memory, or attention) measured by neuropsychological tests or other objective tests. (3) Intervention: any type of exercise training that is structured, planned, repetitive, and purposeful, designed to improve or keep one or more components of physical health. (4) Comparison: no intervention, but routine nursing, sham exercise, health education, or other forms (Caspersen et al. 1985). (5) Outcome: executive function, global cognition, disability, memory function, QoL, and neuropsychiatric symptoms. (6) Study design: RCTs. (7) English‐language studies.

Exclusion criteria: (1) Studies on CI associated with Parkinson's disease, Huntington's disease, multiple sclerosis, epilepsy, diabetes, or psychiatric disorders (often accompanied by various pathological changes besides those directly related to CI, possibly interfering with the efficacy of exercise on cognition). (2) Studies on the effects of acute exercise. If the same or overlapping data arising from the same study were reported across reviews, only the latest ones were included.

### 2.4 Data Extraction

The following data were extracted independently by the two reviewers (QC and HJ): (1) Study characteristics (first author, year of publication, title, and country). (2) Samples (number of participants, sex, and mean age). (3) Interventions. (4) Duration of intervention. (5) Outcome metrics: cognitive function assessed using the Montreal Cognitive Assessment (MoCA) (Jia et al. 2021) and Mini‐mental State Examination (MMSE) (Jannati et al. 2024). When necessary, the reviewer would email the authors to obtain any missing data.

The interventions consisted of Aero, RT, CT, sensorimotor training (ST), mind‐body movement (MB), daily behavior (Daily), routine rehabilitation (RR), and health education (HE). Aero included interventions designed to increase energy expenditure and heart rate, which offered the oxygen consumption required for muscle activation for a sustained period, such as running on a treadmill, bicycling or walking, and interval training. RT was designed to enhance muscle and strength. ST could strengthen neuromuscular connections by balance and coordination. MB focused on breathing and postural relief exercises such as Tai Chi, Baduanjin, and Pilates. CT was defined as a combination of any of the above interventions. Daily had no interventions in daily life.

### 2.5 Quality Assessment

All studies were independently assessed by the two reviewers (QC and HJ) using the Cochrane Rob 1.0 tool (Higgins and Green 2011) from (1) random sequence generation, (2) blinding of participants and personnel, (3) allocation concealment, (4) incomplete outcome data, (5) blinding of outcome assessment, (6) selective reporting, and (7) other bias. Each study was rated as “low,” “high,” or “some concerns.”

### 2.6 Statistical Analysis

RevMan 5.4, Stata15, and R4.4.1 were utilized for NMA. Continuous variables were tested for effect sizes (ESs) by mean difference (MD) with 95% confidence interval (95% Crl). Network evidence maps visualize interventions. The I test was used to analyze the heterogeneity of the study. When I2 ≤ 50%, the fixed effects model was chosen; when I2 > 50%, the random effects model was selected. The Markov Chain Monte Carlo process was utilized for modeling, with four chains running, 20,000 iterations for annealing, and 50,000 iterations for modeling. Moreover, the model fit and global homogeneity were compared by the DIC. In the case of the presence of a closed‐loop network, node‐splitting analysis was performed for regional homogeneity. The surface under the cumulative ranking curve (SUCRA) of interventions ranged from 0% (the least optimal intervention) to 100% (the optimal intervention) (Salanti et al. 2011), and a larger SUCRA corresponded to a higher rank of intervention. When two‐by‐two comparisons of interventions included > 10 articles, publication bias was detected using funnel plots and egger tests.

## 3 Results

### 3.1 Search Results

**FIGURE 1:**
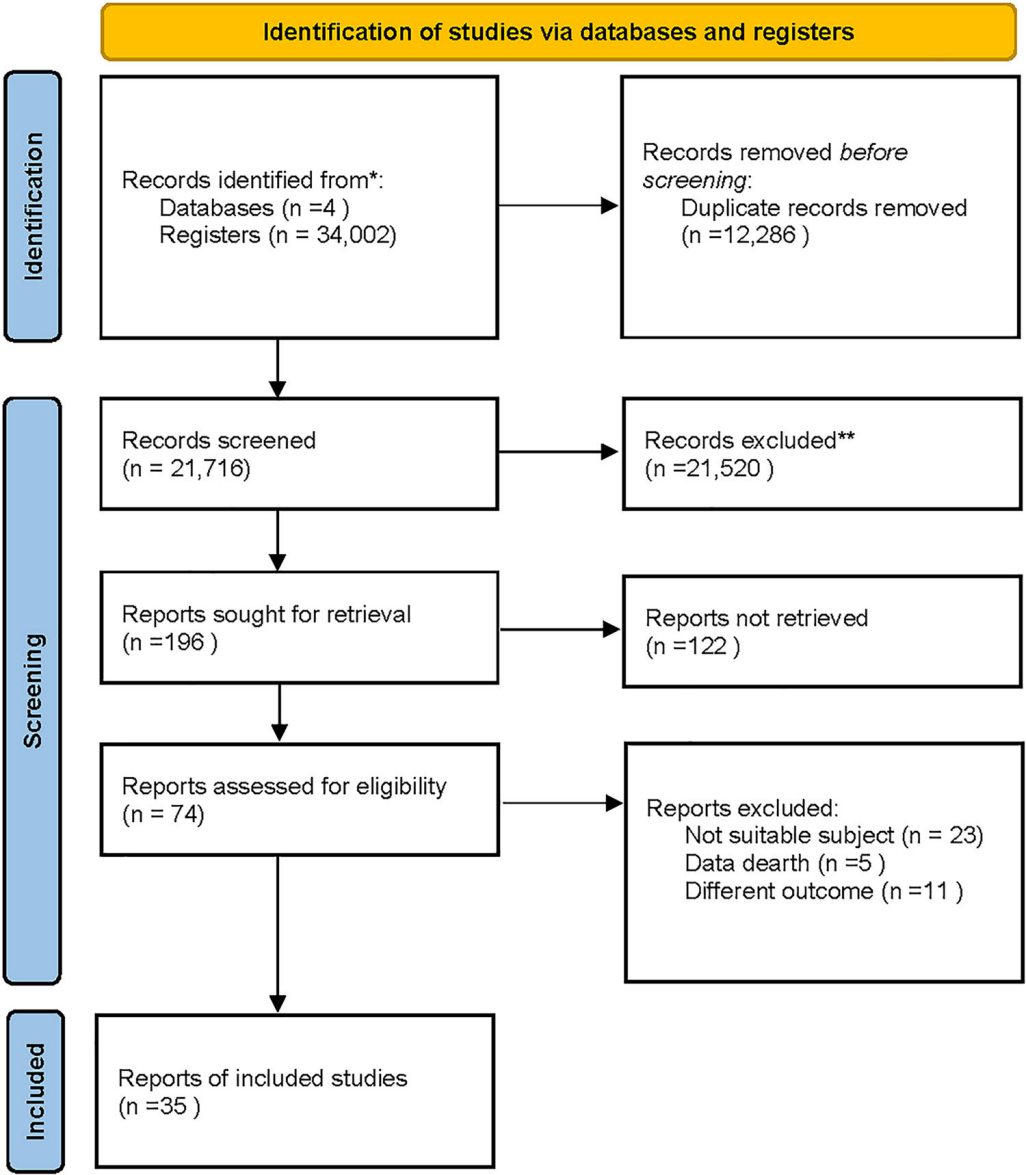
Flowchart.

Initially, 34,002 studies were retrieved using the search strategy. Following the removal of 12,286 duplicate publications, another 21,520 non‐eligible studies were removed by reading the title and abstract (non‐eligible in PICOS). The remaining 196 were reviewed for the full text, of which 122 were excluded due to unavailable full text, 23 due to non‐eligible population, five due to non‐extractable data, and 11 due to non‐eligible outcome metrics. Finally, 35 studies were included (Bisbe et al. 2020; Choi and Lee 2018; Siu and Lee 2018; Bademli et al. 2019; Choi and Lee 2019; Q. Zhang et al. 2023; Yu et al. 2022; Xia et al. 2023; Wei and Ji 2014; L. Wang et al. 2020; Varela et al. 2012; Song and Yu 2019; Rivas‐Campo et al. 2023; Liu et al. 2022; Lazarou et al. 2017; Langoni et al. 2019; Lam et al. 2012; Khattak et al. 2022; Khanthong et al. 2021; Greblo Jurakic et al. 2017; Hong et al. 2018; De Sá et al. 2024; Zhu et al. 2018; Tao et al. 2019; Suzuki et al. 2012; Wang et al. 2024; Buele et al. 2024; Zhou 2025; Baek et al. 2024; Gul Khattak et al. 2024; Krootnark et al. 2024; Sánchez‐Alcalá et al. 2025; Guzel and Can 2024; Tsai et al. 2025; Song et al. 2023) (Figure 1).

### 3.2 Study Characteristics

As shown in Table S1, the included studies were from 13 countries, with 2717 patients aged 60–83 years. Aero was adopted in 18 studies, ST in five studies, MB in nine studies, RT in three studies, and CT in three studies. For the outcome metrics, MoCA and MMSE scores were reported in 23 and 15 studies, respectively.

### 3.3 Quality Assessment

**FIGURE 2:**
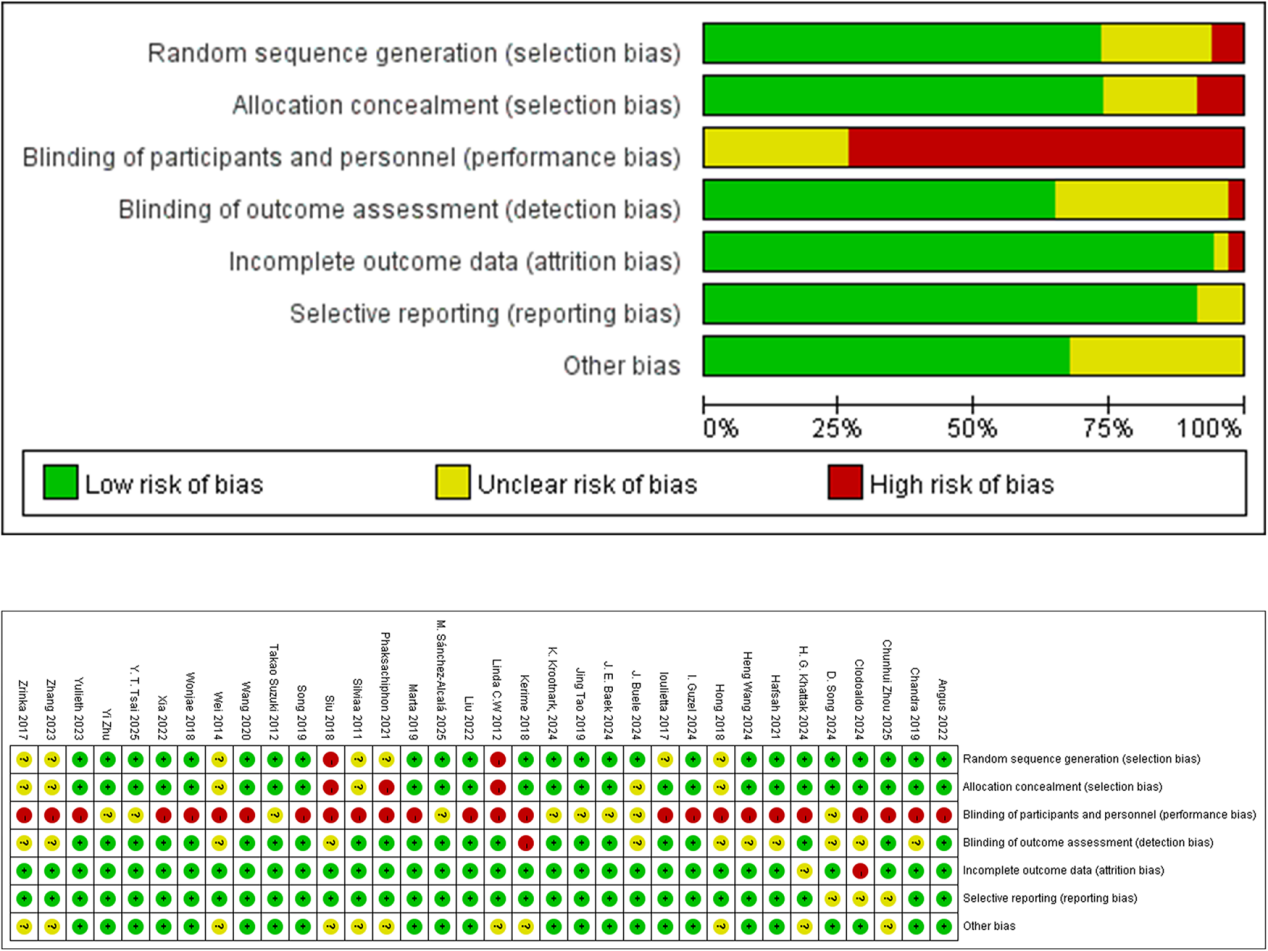
Quality evaluation results.

Using the Cochrane Rob 1.0 tool, we found that seven studies were rated as some concerns and two as high risk (all assigned the appropriate intervention depending on the site where they were located) in random sequence generation; Most of the included studies were rated as having some problems or high risk in the blinded assessment dimension, as it was difficult to conduct the study to implement a double‐blind or triple‐blind study on exercise; in incomplete outcome data, one study was rated as high risk (attrition bias due to quantitative, qualitative, and operational deficiencies in incomplete outcome data); in selective reporting, three studies were considered to have some concerns; and in other bias, 11 studies were considered to have some concerns (Figure 2).

### 3.4 Meta‐Analysis Results

#### 3.4.1 MoCA

**FIGURE 3:**
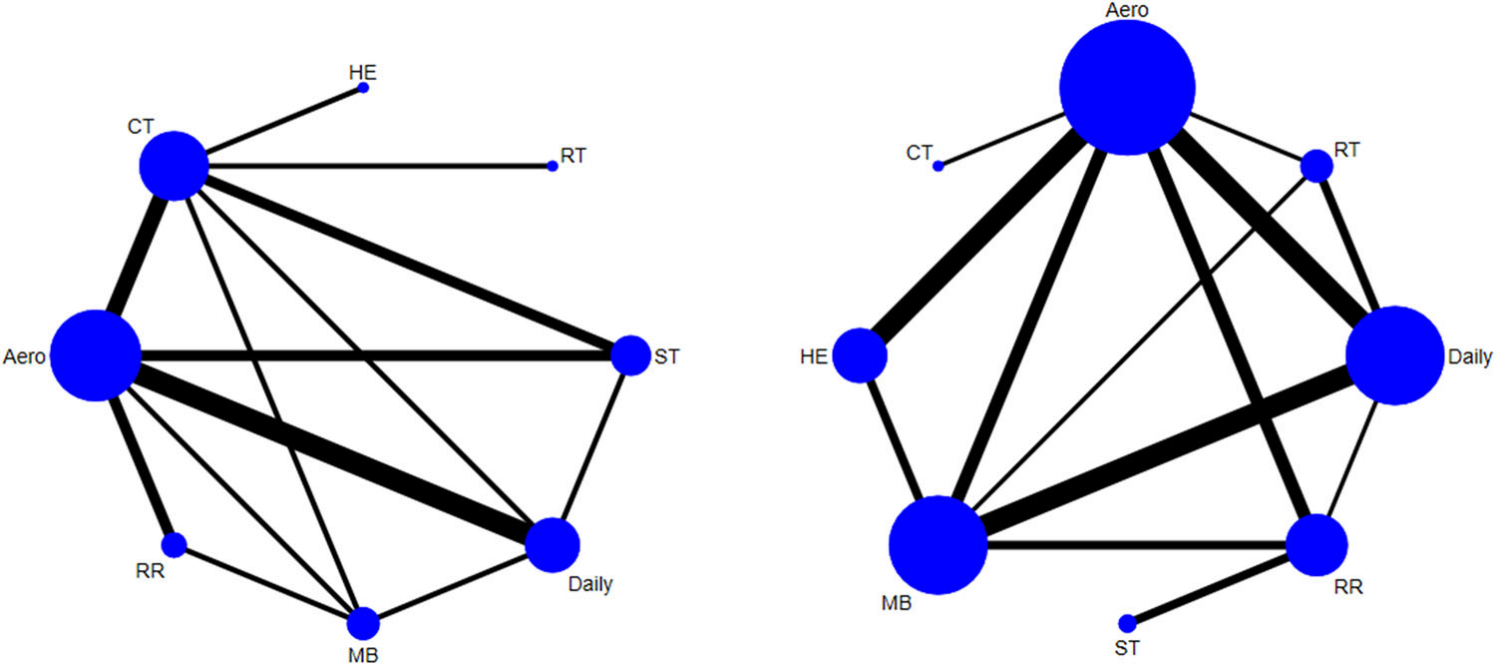
Reticulated evidence map (left; MOCA right MMES).

**FIGURE 4:**
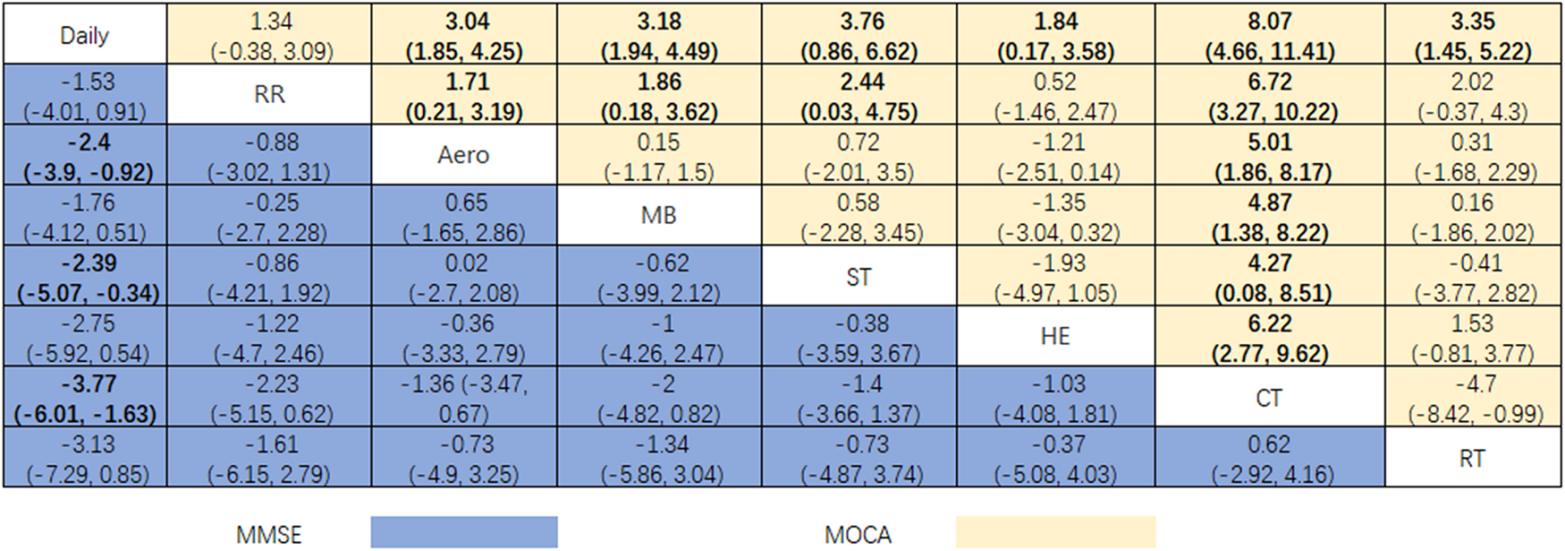
League table.

**FIGURE 5:**
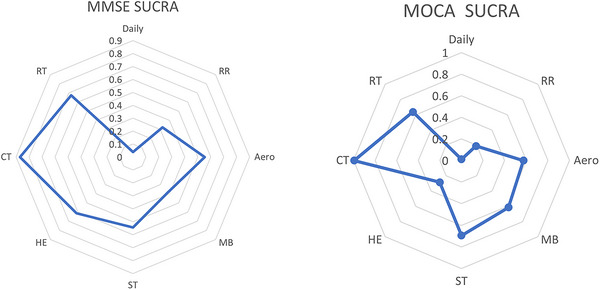
SUCRA results (left MOCA right MMSE).

The MoCA score was reported in 22 studies. A closed loop was found in the network graph (Figure 3), the difference in DIC between the consistent and inconsistent models was < 5, and the local inconsistency results were not statistically significant (*p* > 0.05), indicating the consistency of direct and indirect evidence (Figure S1). As could be seen from the league table (Figure 4), all exercises except RR (MD = 1.34, 95% Crl: −0.38 to 3.09) improved MoCA scores more greatly than Daily. CT was overall superior to others in increasing MoCA scores. Among them, we have Daily (MD = 8.07, 95% Crl: 4.66–11.41), RR (MD = 6.72, 95% Crl: 3.27–10.22), Aero (MD = 5.01, 95% Crl: 1.86–8.22), MB (MD = 4.87, 95% Crl: 1.38–8.22), ST (MD = 4.27, 95% Crl: 0.08–8.51), HE (MD = 6.22, 95% Crl: 2.77–9.62), RT (MD = −4.7, 95% Crl: −8.42 to −0.99). The results of SUCRA showed: CT had the largest (99.43%), followed by ST (69.41%) and RT (63.68%) (Figure 5).

#### 3.4.2 MMSE

The MMSE score was reported in 15 studies. Closed loops were found in the network diagram (Figure 3), and the difference in DIC between consistency and inconsistency models was < 5, and the local inconsistency results were not statistically significant (*p* > 0.05), indicating the consistency of direct and indirect evidence (Figure S2). As could be seen from the league table (Figure 4), Aero (MD = −2.4, 95% Crl: −3.9 to −0.92), ST (MD = ‐2.39, 95% Crl: −5.07 to −0.34), and CT (MD = −3.77, 95% Crl: −6.01 to −0.163) could significantly ameliorate the cognitive function in MCI patients compared with Daily, with CT having the largest ES (Figure 3). The results of SUCRA showed that CT had the largest (87.04%), followed by RT (67.35%) and HE (61.41%) (Figure 5).

## 4 Discussion

Thirty‐five RCTs with 2717 MCI patients were included, involving 23 different exercises (eight types of interventions). The NMA results revealed that CT greatly improved or relieved MCI from the perspective of MoCA or MMSE, and it was also identified as the optimal intervention, followed by RT and ST. In several recent NMAs on nonpharmacological interventions (Bruderer‐Hofstetter et al. 2018; Huang et al. 2022), exercise is recommended as a means for ameliorating MCI (Song and Yu 2019), which plays an increasingly important role in preventing cognitive decline and improving QoL of CI patients (Tomoto et al. 2021; Raichlen et al. 2020).

The capacity of CT to ameliorate cognitive function and delay progression in MCI patients has been documented in several studies, and it may work by facilitating both nervous system function and neuroplasticity (Buele and Palacios‐Navarro 2023; Chapman et al. 2015). Plenty of physical exercise can contribute to the release of brain‐derived neurotrophic factors, thereby enhancing neuronal survival and growth; by increasing cerebral blood flow and promoting the supply of oxygen and nutrients to the brain, it also creates a more favorable environment for neuronal growth Liao et al. (2020). Doniger et al. (2018) found that cognitive training can activate the prefrontal lobe, middle cingulate, and posterior cingulate cortex and increase cerebral blood flow in these regions, thereby effectively improving the metabolic activity and neural function of the brain. An increase in cerebral blood flow is usually accompanied by an improvement in brain activity, during which neuroplasticity may be altered, so that the brain becomes more adaptive to cognitive challenges (Yamada and Sumiyoshi 2021). Such a mechanism has also been demonstrated in animal experiments (Mora 2013). In addition, more evidence is presented by neuroimaging studies that CT more significantly increases blood flow in the prefrontal lobe, hippocampus, and cerebellum than any single training, suggesting that CT may exert a more comprehensive and synergistic effect on multiple cognitive brain regions (Doniger et al. 2018). Park further demonstrated that CT can stimulate the hypothalamic‐pituitary‐adrenal axis to regulate the activity of the neuroendocrine system while enhancing learning and memory functions, which is a physiological basis for cognitive enhancement. Besides, ST can mobilize the patient's capacity for postural control and spatial orientation, and improve the brain's capacity for real‐time prediction and adjustment of movement by activating the cerebellar‐vestibular‐cortical network; in this way, MCI patients can suffer less “distraction” and possess stronger cognitive flexibility, attention, and spatial awareness (Antonioni et al. 2024; Rogge et al. 2018). In particular, patients receiving complex CT need to simultaneously handle multiple tasks of exercise or in a specific order, during which they have to allocate and deploy limited attention resources, thus activating the synergy of multimodal neural circuits and further optimizing the multitasking ability. This multitasking training can help the brain coordinate various functional regions and possess better overall cognitive performance in response to different cognitive demands.

Jun Hwan Choi also offered robust evidence on motor control and cognitive function (Choi et al. 2015). In stroke patients, CT not only improves dynamic balance parameters but also greatly strengthens short‐term visuospatial memory and auditory attention, indicating that motor and cognitive functions share neural resources in the brain. Such synergy between the two is further validated by the co‐activation of prefrontal executive and sensorimotor networks, which may ameliorate long‐term cognitive function by enhancing the plasticity and efficiency of neural networks. CT also achieves the same effect on the healthy elderly. In addition, continuous meditation training can modulate the resting‐state activity of the default mode network (DMN), help patients stay focused, and enhance the anti‐correlation connectivity between task‐positive networks and DMN. This mechanism not only effectively reduces DMN hyperactivation commonly seen in MCI patients but also ameliorates their cognitive function (Ramírez‐Barrantes et al. 2019). Since DMN hyperactivation is closely linked to cognitive decline in MCI and early AD, CT may exert a key intervention effect in the early stage of disease.

Some limitations are worth noting. First, since cognitive defects (deficits in memory and/or other cognitive domains, but not necessarily in two or several domains) greatly vary across MCI classifications, MCI patients were not further subdivided; few studies were available on each domain, which precluded subgroup analyses on MCI, making it difficult to precisely clarify the effects of different modes of exercise on each functional region. Second, the influence of duration and frequency of training on individuals is still not fully understood, so future studies can explore how to optimize these factors. Finally, only English‐language studies were included, which may result in missing data.

## 5 Conclusion

This NMA synthesized the existing evidence from previous studies and presented important findings on exercise therapy for clinical practitioners and investigators. In conclusion, CT is the optimal intervention for increasing MoCA and MMSE scores, and it is most likely to ameliorate MCI. In the future, higher‐quality, multicenter, large‐sample RCTs are required to further validate these findings.

## Author Contributions

**Changjiang Qiu**: writing – review and editing, writing – original draft, conceptualization, methodology, formal analysis, investigation. **Jiaxin Han**: formal analysis, investigation. **Yao Xie**: supervision. **Lu Liu**: investigation, formal analysis. **Yufeng Zeng**: formal analysis, investigation. **Liangyi Xiao**: supervision. **Shanshan Zeng**: supervision. **Le Xie**: supervision. **Dahua Wu**: funding acquisition, resources.

## Conflicts of Interest

The authors declare no conflicts interest.

## Peer Review

The peer review history for this article is available at https://publons.com/publon/10.1002/brb3.70734

## Supporting information




**Supporting Fig.1**: Consistency test (MOCA)

**Supporting Fig.2**: Consistency test (MMSE)

**Supporting Table 1**: Characteristics of included studies

## Data Availability

The datasets used and/or analyzed during the current study available from the corresponding author on reasonable request.

## SEARCH STRATEGY (PUBMED)

**Table:**

Pubmed		
	Query	Results
1	Cognitive Dysfunction[MeSH Terms]	45,872
2	(((((((((Cognitive Dysfunction[Title/Abstract]) OR (mild Cognitive Dysfunctions[Title/Abstract])) OR (mild Cognitive Impair*[Title/Abstract])) OR (mild Cognitive Disorder*[Title/Abstract])) OR (mild cogniti* disorder*[Title/Abstract])) OR (mild Cogniti* Declin*[Title/Abstract])) OR (mild Mental Deterioration*[Title/Abstract])) OR (mild cogniti* defect*[Title/Abstract])) OR (mild cogniti* deteriorat*[Title/Abstract])) OR (mild cogniti* decrement*[Title/Abstract])	51,610
3	(“Cognitive Dysfunction”[Mesh]) OR ((((((((((Cognitive Dysfunction[Title/Abstract]) OR (mild Cognitive Dysfunctions[Title/Abstract])) OR (mild Cognitive Impair*[Title/Abstract])) OR (mild Cognitive Disorder*[Title/Abstract])) OR (mild cogniti* disorder*[Title/Abstract])) OR (mild Cogniti* Declin*[Title/Abstract])) OR (mild Mental Deterioration*[Title/Abstract])) OR (mild cogniti* defect*[Title/Abstract])) OR (mild cogniti* deteriorat*[Title/Abstract])) OR (mild cogniti* decrement*[Title/Abstract]))	77,726
4	Exercise[MeSH Terms]	267,988
5	((((((((((((((((((exercise training[Title/Abstract]) OR (resistance training[Title/Abstract])) OR (whole body vibration[Title/Abstract])) OR (WBV[Title/Abstract])) OR (Low Intensity Vibration[Title/Abstract])) OR (vibration[Title/Abstract])) OR (oscillating platforms[Title/Abstract])) OR (aerobic training[Title/Abstract])) OR (walking[Title/Abstract])) OR (physical activity[Title/Abstract])) OR (highimpact exercise[Title/Abstract])) OR (Endurance Training[Title/Abstract])) OR (Strength Training[Title/Abstract])) OR (activity[Title/Abstract])) OR (exercise[Title/Abstract])) OR (Training[Title/Abstract])) OR (workout[Title/Abstract])) OR (dance[Title/Abstract])) OR (Tai Chi Chuan[Title/Abstract])	4,240,032
6	(“Exercise”[Mesh]) OR (((((((((((((((((((exercise training[Title/Abstract]) OR (resistance training[Title/Abstract])) OR (whole body vibration[Title/Abstract])) OR (WBV[Title/Abstract])) OR (Low Intensity Vibration[Title/Abstract])) OR (vibration[Title/Abstract])) OR (oscillating platforms[Title/Abstract])) OR (aerobic training[Title/Abstract])) OR (walking[Title/Abstract])) OR (physical activity[Title/Abstract])) OR (highimpact exercise[Title/Abstract])) OR (Endurance Training[Title/Abstract])) OR (Strength Training[Title/Abstract])) OR (activity[Title/Abstract])) OR (exercise[Title/Abstract])) OR (Training[Title/Abstract])) OR (workout[Title/Abstract])) OR (dance[Title/Abstract])) OR (Tai Chi Chuan[Title/Abstract]))	4,291,667
7	‘randomized controlled trial’ OR ‘controlled clinical trial’ OR ‘randomized’ OR ‘randomised’ OR ‘placebo’ OR ‘drug therapy’ OR ‘randomly’ OR ‘trial’ OR ‘groups’ NOT ‘animals’	8,323,895
8	(#1 OR #2 OR #3) AND (#4 OR #5 OR #6) AND #7	5,513
