# Supplementary figures and images for: Comparison of the Efficacy of Different Exercise Modes on MCI Adults: A Network Meta‐Analysis
Source: Brain Behav. 2025 Aug 21;15(8):e70734. doi: 10.1002/brb3.70734 (PMC12370853; doi:10.1002/brb3.70734)

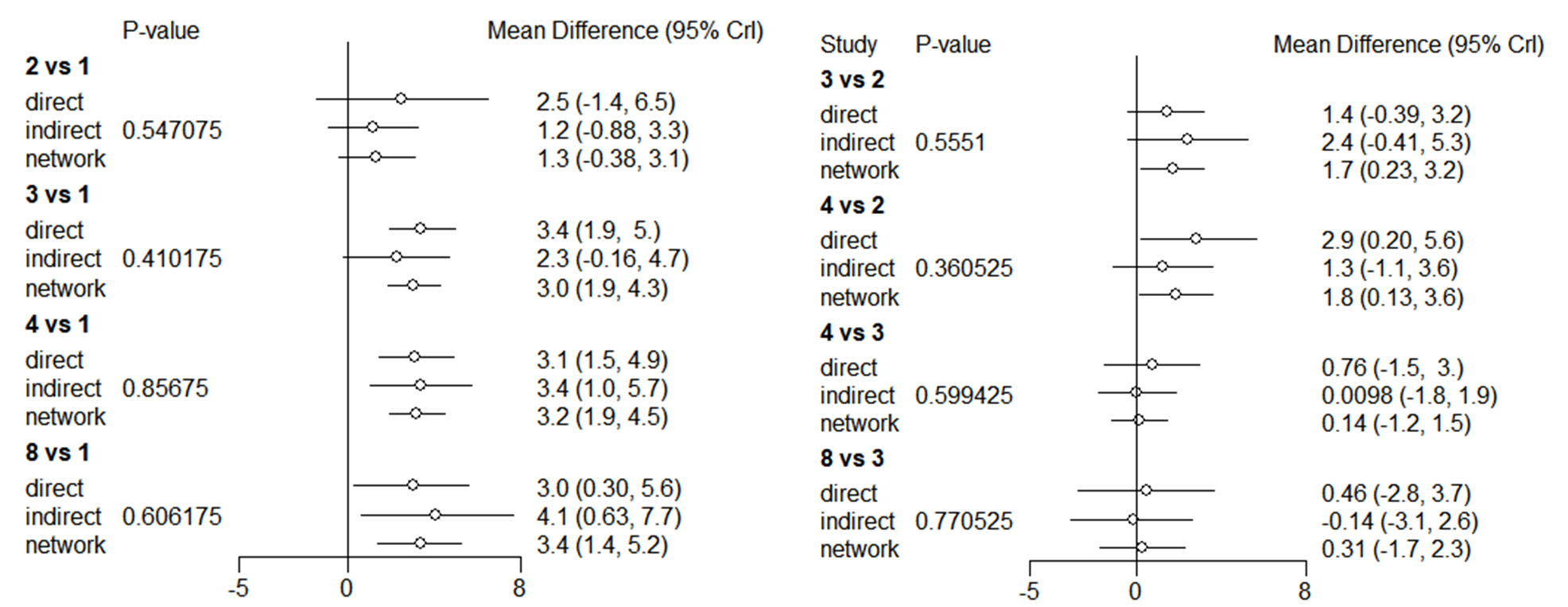

Supplement: Supplementary file 1 — Supporting Fig.1: Consistency test (MOCA) [file BRB3-15-e70734-s002.tif]

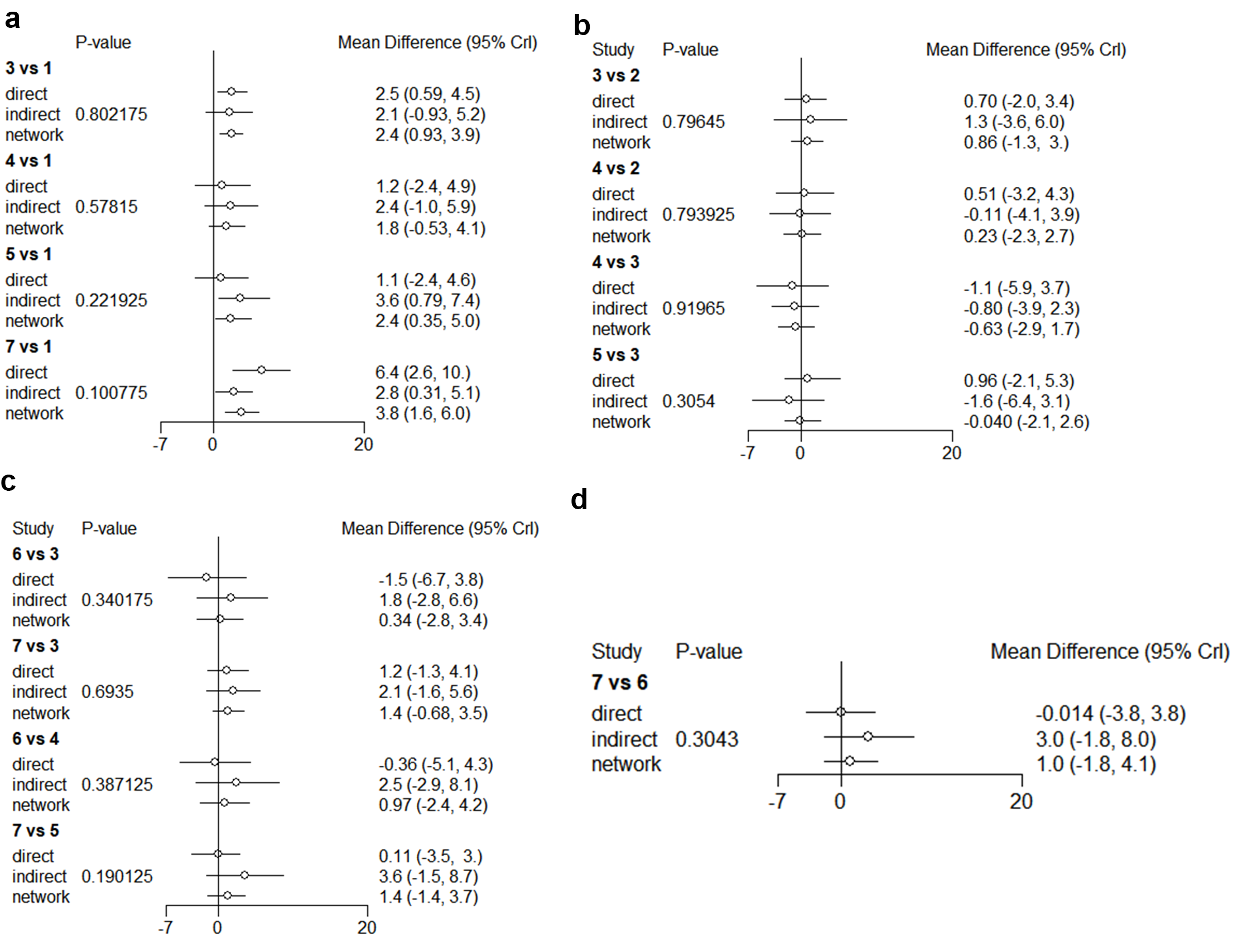

Supplement: Supplementary file 2 — Supporting Fig.2: Consistency test (MMSE) [file BRB3-15-e70734-s001.tif]
