# Supplementary material for: Comparison of the Efficacy of Different Exercise Modes on MCI Adults: A Network Meta‐Analysis
Source: Brain Behav. 2025 Aug 21;15(8):e70734. doi: 10.1002/brb3.70734 (PMC12370853; doi:10.1002/brb3.70734)
Supplement: Supplementary file 3 — Supporting Table 1: Characteristics of included studies [file BRB3-15-e70734-s003.docx]

**Table S1** Characteristics of included studies

| **Study** | **Country** | **Design** | **People** | **Sample** | **Age(IG/CG)** | **Gender(F/M)** | **Outcome** |
| --- | --- | --- | --- | --- | --- | --- | --- |
| Marta et al.,2019 | Spain | RCT | MCI | 60 | 72.88 ± 5.60 77.29 ± 5.16 | 16/15 | MMSE |
| Wonjae et al.,2018 | Korea | RCT | MCI | 60 | 74.90 ± 5.10 74.23 ± 4.38 | 49/11 | MoCA |
| Siu et al.,2018 | China | RCT | MCI | 240 | not reported | 178/62 | MMSE |
| Kerime et al.,2018 | Turkey | RCT | MCI | 60 | 72.24 ± 7.16 70.67 ± 8.34 | 35/25 | MMSE |
| Wonjae et al.,2018 | Korea | RCT | MCI | 60 | 77.2 7± 4.37 75.37 ± 3.97 | 51/9 | MoCA |
| Zhang et al.,2023 | China | RCT | MCI | 42 | 66.67 ± 6.04 66.22 ± 5.51 69.75 ± 7.02 | 38/4 | MoCA,MMSE |
| Angus et al.,2022 | China | RCT | MCI | 34 | 67.3 ± 4.2 67.2 ± 6.8 67.6 ± 8.1 | 25/9 | MoCA |
| Xia et al.,2022 | China | RCT | MCI | 135 | 66.16 ± 4.16 66.08 ± 4.28 65.41 ± 4.90 | 85/50 | MoCA |
| Wei et al.,2014 | China | RCT | MCI | 60 | 66.73 ± 5.48 65.27 ± 4.63 | 20/40 | MMSE |
| Wang et al.,2020 | China | RCT | MCI | 111 | 68.37 ± 5.27 68.24 ± 5.15 | 68/43 | MoCA |
| Silviaa et al.,2011 | Spain | RCT | MCI | 48 | 79.24 ± 10.07 76.44 ± 11.38 79.40 ± 6.72 | 21/27 | MMSE |
| Song et al.,2019 | China | RCT | MCI | 120 | 76.22 ± 5.76 75.33 ± 6.78 | 90/30 | MoCA |
| Yulieth et al.,2023 | Colombia | RCT | MCI | 132 | 77.11 ± 7.3 77.19 ± 7.7 | 79/53 | MoCA |
| Liu et al.,2022 | China | RCT | MCI | 50 | 74.6 ± 6.1 73.2 ± 6.3 73.4 ± 6.5 | 35/15 | MoCA |
| Ioulietta et al.,2017 | Greece | RCT | MCI | 129 | 65.89 ± 10.76 67.92 ± 9.47 | 101/28 | MoCA,MMSE |
| Chandra et al.,2019 | Brazil | RCT | MCI | 52 | 72.6 ± 7.8 71.9 ± 7.9 | 40/12 | MMSE |
| Linda C.W. et al.,2012 | China | RCT | MCI | 389 | 77.2 ± 6.3 78.3 ± 6.6 | 297/92 | MMSE |
| Hafsah et al.,2021 | Pakistan | RCT | MCI | 59 | 62.49 ± 1.82 | 24/35 | MoCA,MMSE |
| Phaksachiphon et al.,2021 | Thailand | RCT | MCI | 71 | 60.26 ± 5.67 61.47 ± 7.49 | 56/15 | MoCA |
| Zrinka et al.,2017 | Croatia | RCT | MCI | 28 | 70.4 ± 3.93 | 28(Female) | MoCA |
| Hong et al.,2018 | Korea | RCT | MCI | 22 | 75.53 ± 4.48 | 16/6 | MoCA |
| Clodoaldo et al.,2024 | Brazil | RCT | MCI | 27 | 70.0 ± 6.7 73.6 ± 7.4 70.3 ± 5.6 | 21/6 | MMSE |
| Buele, Jorge et al. 2024 | Ecuador | RCT | MCI | 34 | 77.35±6.75 75.41±5.76 | 23/11 | MOCA |
| Zhou, Chunhui 2025 | China | RCT | MCI | 66 | 66.9±4.94 66.05±6.64 64.58±4.86 | 50/16 | MOCA |
| Guzel, Ilkem et al.2024 | Turkiye | RCT | MCI | 31 | 82.3±7.8 82.3±6.7 | 10（Female） | MMSE |
| Song, Dan et al 2024 | China | RCT | MCI | 89 | 76.71±5.96 75.20±6.63 | 90/30 | MOCA |
| Baek, Ji-Eun et al 2024 | Korea | RCT | MCI | 44 | 82.40±4.46 81.04±4.93 | 29/15 | MMSE |
| Tsai, Yu-Ting et al. 2025 | China | RCT | MCI | 24 | 78.50±3.40 79.42±7.04 | 3/21 | MOCA |
| Sánchez-Alcalá et al. 2025 | Spain | RCT | MCI | 92 | 71.43±2.97 72.24±2.92 | 34/58 | MMSE |
| Kitsana et al 2024 | Thailand | RCT | MCI | 90 | 69.00±5.03 | 71/19 | MOCA |
| Hafsah Gul Khattak et al.2024 | Pakistan | RCT | MCI | 51 | 62.74±7.4 | 26/25 | MOCA |
| Wang, Heng et al 2024 | China | RCT | MCI | 60 | not reported | not reported | MOCA |
| Zhu, Yi et al. 2018 | China | RCT | MCI | 60 | 70.3±6.7 69.0±7.3 | 36/24 | MOCA |
| Tao, Jing et al. 2019 | China | RCT | MCI | 57 | 66.17±4.17 64.32±2.60 65.97±5.66 | 18/39 | MOCA |
| Suzuki, Takao et al 2012 | Japan | RCT | MCI | 50 | 75.3±7.5 76.8±6.8 | 17/33 | MMSE |
